# Venlafaxine as single therapy associated with hypertensive encephalopathy

**DOI:** 10.1186/s40064-015-0883-0

**Published:** 2015-02-26

**Authors:** Bengt Edvardsson

**Affiliations:** Department of Clinical Sciences, Lund University, Lund, S-221 85 Sweden; Neurology, Skane University Hospital, Lund, S-221 85 Sweden

**Keywords:** Venlafaxine, Hypertensive encephalopathy, Neuroimaging, MRI, Posterior reversible encephalopathy syndrome, PRES, Blood pressure

## Abstract

**Introduction:**

Hypertensive encephalopathy with the clinicoradiological entity posterior reversible encephalopathy syndrome in the setting of venlafaxine as single therapy has not been reported earlier.

**Case description:**

A 46-year-old man developed hypertensive encephalopathy associated with venlafaxine as single therapy. Magnetic resonance imaging of the brain, pre and post gadolinium, carried out on day 2, displayed an increased T2 signal in the cortex on both the T2 and FLAIR images throughout the frontal and temporal lobes and in the cerebellum. Venlafaxine therapy was stopped. The patient gradually improved and he became seizure free and the blood pressure successively became normal. A magnetic resonance imaging after six weeks displayed marked regression of the abnormalities. On follow-up after 3 months, his blood pressure had been normal and he had not had any symptoms. The prescribed antiepileptic drug was discontinued as well as antihypertensive treatment. He had not experienced any new symptoms at follow-up after one year.

**Discussion and evaluation:**

The patient in this report had hypertensive encephalopathy associated with venlafaxine therapy. The imaging findings are compatible with hypertensive encephalopathy/posterior reversible encephalopathy syndrome. Venlafaxine is a drug used very frequently. Venlafaxine may infrequently induce hypertensive crisis.

**Conclusion:**

Hypertensive encephalopathy may rarely occur in the setting of venlafaxine as single therapy even in low to moderate doses. Patients on venlafaxine should have regular monitoring of blood pressure. Knowledge of the side effects is vital. Venlafaxine must be discontinued if significant hypertension persists.

## Background

Depression is usually associated with low systolic blood pressure and less hypertension although medicines intended for treatment of the same disorder can be associated with an increase in blood pressure. Some antidepressants are associated with both high diastolic and systolic blood pressures and hypertension (Licht et al. 2009). Antidepressants are often used for long periods and it is important to clarify any side effects of the treatment. Venlafaxine is an antidepressant, belonging to the serotonin-norepinephrine reuptake inhibitor class of drugs, used for the therapy of depression and anxiety disorders. It is also used (although not approved) as a treatment for diabetic neuropathy and in migraine prophylaxis. The drug is usually well tolerated. Side effects include headache, dizziness, tremor, somnolence, nausea and hypertension. Hypertensive encephalopathy with the clinicoradiological entity posterior reversible encephalopathy syndrome (PRES) in the setting of venlafaxine as single therapy has not been reported earlier. I here describe a 46-year-old man who developed hypertensive encephalopathy associated with venlafaxine as single therapy.

## Case description

**Figure 1:**
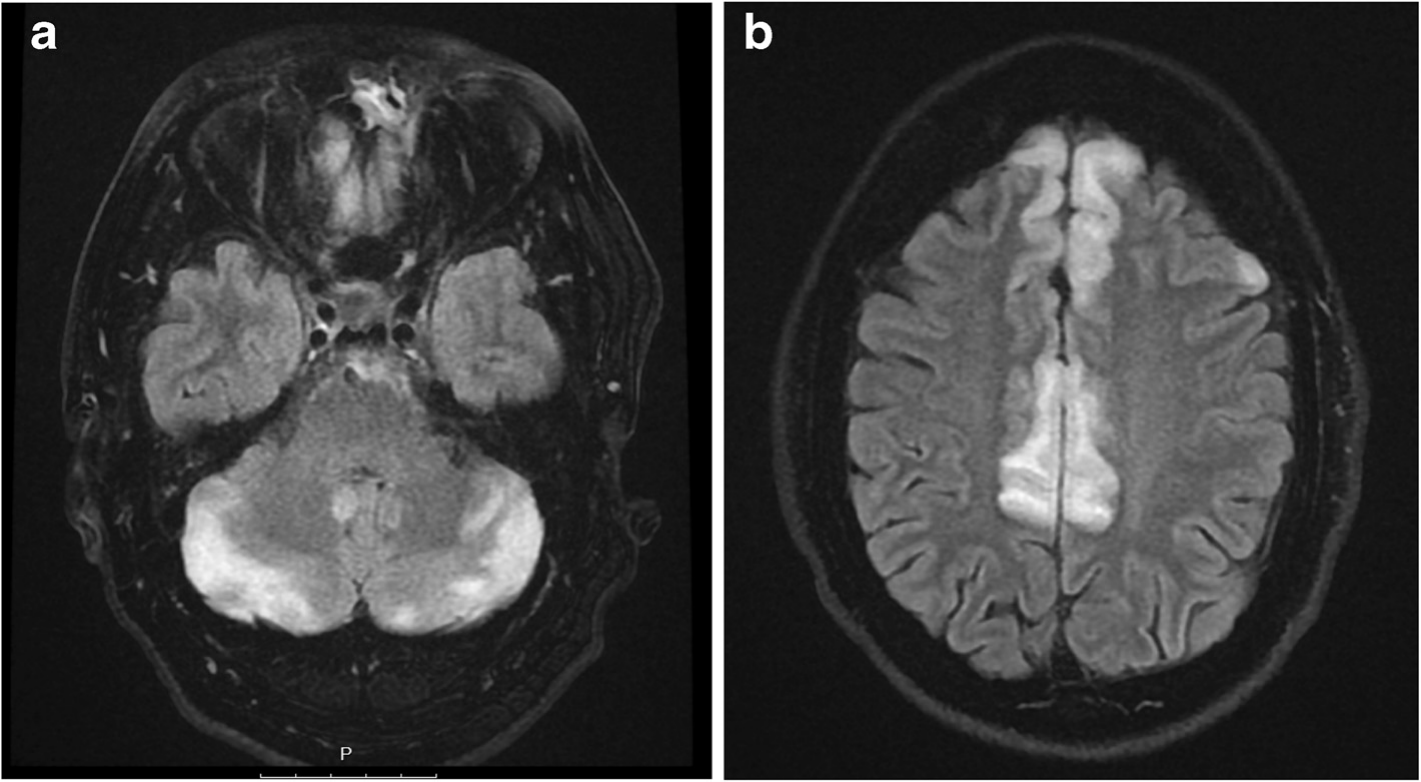
**Primary MRI T2 and FLAIR images a and b displaying an increased signal in the cortex throughout the frontal and temporal lobes and in the cerebellum consistent with hypertensive encephalopathy/PRES.**

**Figure 2:**
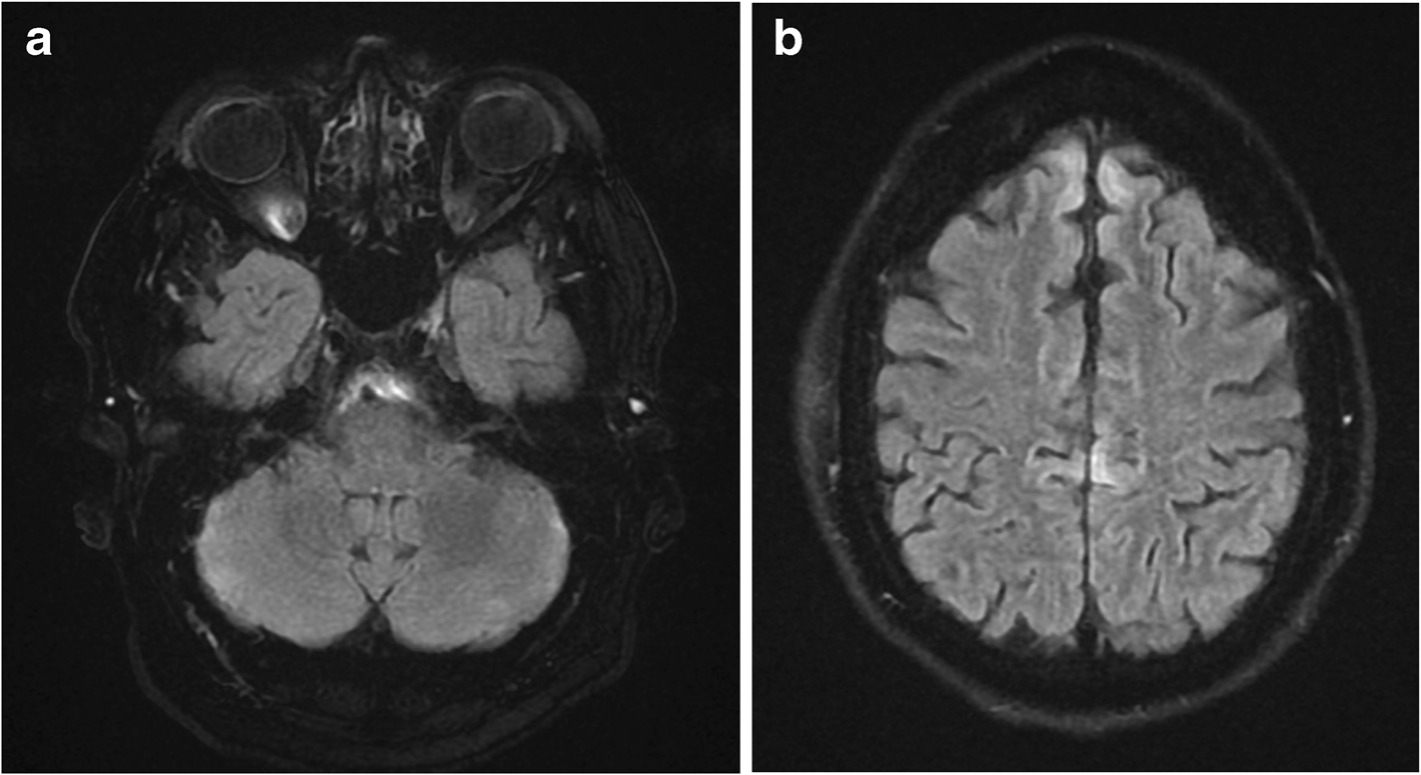
**MRI after 6 weeks a and b displaying marked regression of the abnormalities.**

A 46-year-old man presented with generalized seizures. He was on venlafaxine therapy for depression and anxiety disorder. He had taken venlafaxine for sex weeks. He started on 75 mg/day which was increased to 150 mg/day after two weeks. He was told to continue the medication in that dose. Blood pressure was monitored and he was all the time normotensive. There was no history of seizures, hypertension or other diseases. He had no other medications. The seizures were followed by nausea, vomiting, severe headache, visual disturbances and confusion. He also experienced severe shortness of breath and began to cough up frothy pink sputum. On presentation in the emergency department, he was sleepy, had a pulse of 135 beats/min, a blood pressure of 231/133 mm Hg, and a respiratory rate of 35-38. His chest X-ray indicated pulmonary oedema. He was treated for acute congestive heart failure due to severe hypertension. The hypertension was treated with labetalol and enalaprilat intravenously. Venlafaxine therapy was stopped. Complete blood picture, and blood biochemistry were normal. On day 2 of admittance he experienced further seizures. Treatment with phenytoin and levetiracetam intravenously proved to be effective and the seizures stopped. An electroencephalogram showed diffuse slowing indicating an encephalopathy. Cerebrospinal examination was normal. Echocardiography was normal. Magnetic resonance imaging (MRI) of the brain, pre and post gadolinium, carried out on day 2, displayed an increased T2 signal in the cortex on both the T2 and FLAIR images throughout the frontal and temporal lobes and in the cerebellum consistent with hypertensive encephalopathy/PRES (Figure 1 a and b). No contrast enhancement was seen and diffusion weighted imaging showed no signs of ischemia. The mental status changes improved after treatment. Oral anticonvulsive and antihypertensive treatment continued. Other secondary causes of hypertension were excluded. The patient gradually improved and he became seizure free and the blood pressure successively became normal. Physical examination showed no signs of heart disease. Repeated chest X-ray was normal. The patient was discharged to his home on day 12. A MRI after six weeks displayed marked regression of the abnormalities (Figure 2 a and b). The patient was seen again after three months. The prescribed antiepileptic drug was discontinued as well as antihypertensive treatment. His blood pressure had been normal and he had not had any seizures. He denied any cardiac symptoms. He had not experienced any new symptoms at follow-up after one year. He is recurrently evaluated by psychiatrist. He now uses a different medicine for his depression and anxiety disorder.

## Discussion

The case study highlights a patient with hypertensive encephalopathy in the setting of venlafaxine as single therapy. The patient had no earlier known hypertension and he recovered completely after discontinuation of venlafaxine. Consequently, venlafaxine was the only rational cause of the hypertensive encephalopathy in this case.

Hypertension is a known side effect of venlafaxine therapy. However, the incidence of venlafaxine induced high blood pressure is low and the effect on blood pressure is weak (Lacy et al. 2003). (Thase 1998) found in a study of venlafaxine diastolic blood pressure elevations only in 4,8% of patients. The mean rise was only 1, 02 mmHg. Hypertensive crisis was not observed. (Fabre & Putman 1987) observed a rise in systolic blood pressure after administration of a125 to 250 mg dose of venlafaxine in healthy volunteers. In a study with venlafaxine, (Schweizer et al. 1991) found an elevation of diastolic blood pressure of 5, 6 mmHg versus baseline. (Chugh et al. 2013) observed in a study of venlafaxine and fluoxetine a significant rise in blood pressure which was in line with earlier published studies (Licht et al. 2009; Thase 1998). The rise of blood pressure is dose dependent, happening more often at higher doses (Thase 1998). The half-life of venlafaxine is relatively short, which explains the generally rapid normalization of blood pressure after discontinuation of venlafaxine (Lacy et al. 2003). The mechanism behind venlafaxine induced hypertension is unclear. The hypertension may be caused by noradrenergic potentiation by venlafaxine (Sawynok et al. 2001). To my knowledge, venlafaxine induced acute hypertension is earlier only reported in two case reports. (Pardal et al. 2001) reported a case of high blood pressure associated with venlafaxine. The dose of venlafaxine was 150 mg/day and blood pressure was 162/110 mm Hg. However, no signs of hypertensive emergency/encephalopathy were noticed. Venlafaxine was stopped and the patient became normotensive. In another study (Khurana & Baudendistel 2003) described a case with hypertensive crisis associated with venlafaxine. Blood pressure was 224/148 mm Hg. The dose of venlafaxine was only 75 mg/day but that patient also took disulfiram which may have increased the toxicity of venlafaxine. Disulfiram as single therapy can also cause hypertension (Khurana & Baudendistel 2003). A MRI of the brain displayed abnormalities consistent with hypertensive encephalopathy/PRES. After blood pressure treatment and discontinuing of venlafaxine the patient improved and became normotensive and discharged on day 3.

However, it must be emphasized that the risk of hypertensive crisis during treatment with venlafaxine is low. Venlafaxine is a drug used very frequently. Only two case reports in the literature describing hypertensive crises during medication of venlafaxine have been published. Thus, the evidence is weak. Hypertensive crisis could also be a confounding factor. Additional reports are needed to strengthen the association between venlafaxine and hypertensive crisis.

## Conclusions

Hypertensive encephalopathy may rarely occur in the setting of venlafaxine as single therapy even in low to moderate doses. Patients on venlafaxine should have regular monitoring of blood pressure. Knowledge of the side effects is vital. Venlafaxine must be discontinued if significant hypertension persists.

## Consent

Informed consent from the patient for the case report to be published.

## References

[CR1] Chugh PK, Kalra BS, Kaushik N, Tekur U (2013). Evaluation of anti-inflammatory activity, effect on blood pressure & gastric tolerability of antidepressants. Indian J Med Res.

[CR2] Fabre LF, Putman HP (1987). An ascending single-dose tolerance study of WY45030, a bicyclic antidepressant, in healthy men. Curr Ther Res.

[CR3] Khurana RN, Baudendistel TE (2003). Hypertensive crisis associated with venlafaxine. Am J Med.

[CR4] Lacy CF, Armstrong LL, Goldman MP, Lance LL (2003). Drug Information Handbook.

[CR5] Licht CM, de Geus EJ, Seldenrijk A, van Hout HP, Zitman FG, van Dyck R, Penninx BW (2009). Depression is associated with decreased blood pressure, but antidepressant use increases the risk for hypertension. Hypertension.

[CR6] Pardal PK, John TR, Rathee SP (2001). Venlafaxine induced hypertension: a case report. Indian J Psychiatry.

[CR7] Sawynok J, Esser MJ, Reid AR (2001). Antidepressants as analgesics: an overview of central and peripheral mechanisms of action. J Psychiatry Neurosci.

[CR8] Schweizer E, Weise C, Clary C, Fox I, Rickels K (1991). Placebo-controlled trial of venlafaxine for the treatment of major depression. J Clin Psycho Pharmacology.

[CR9] Thase ME (1998). Effects of venlafaxine on blood pressure (a meta-analysis of original data from 3744 depressed patients). J Clin Psychiatry.

